# Multicentre study on dynamic contrast computed tomography findings of focal liver lesions with clinical and histological correlation

**DOI:** 10.4102/sajr.v23i1.1667

**Published:** 2019-05-21

**Authors:** Sheila T. Ominde, Timothy M. Mutala

**Affiliations:** 1 Department of Diagnostic Imaging and Radiation Medicine, University of Nairobi, Nairobi, Kenya

**Keywords:** Dynamic contrast CT, focal liver lesion, enhancement pattern, arterial phase, porto-venous phase, delayed phase

## Abstract

**Background:**

Current advancements in dynamic contrast imaging of the liver have enabled increased sensitivity in the diagnosis of liver lesions. Evaluation and characterisation of the enhancement pattern of liver lesions in respect to the liver parenchyma aids in making a specific diagnosis.

**Objectives:**

The aim of this study was to determine the liver findings on dynamic contrast computed tomography (CT) scanning and correlate them with clinicopathologic findings.

**Methods:**

This prospective cross-sectional study included 61 patients and took place between August 2017 and February 2018. Dynamic contrast CT was performed and the images were evaluated by two experienced radiologists. Correlation of the CT findings with histology results from an ultrasound-guided biopsy was done. Data analysis was performed using SPSS version 20.0.

**Results:**

Hepatocellular carcinoma (HCC) was the most common malignant lesion seen and showed three patterns of enhancement: homogenous, abnormal internal vessels and heterogeneous enhancement. Abnormal internal vessel pattern was most specific (90.6%) and showed a high positive predictive value (PPV) of 78.6%. Rapid washout showed a specificity of 87.5% and a PPV of 72.2% in the diagnosis of HCC. Dynamic contrast CT scan had a sensitivity of 93%, specificity of 50%, PPV of 91% and diagnostic accuracy of 95.5% in differentiation of benign and malignant liver lesions. Considering only Liver Imaging Reporting and Data System (LI-RADS) category 5 as conclusive for HCC diagnosis, our study did not miss a significant number of HCCs. Liver Imaging Reporting and Data System category 5 showed specificity of 81.3% and PPV of 75%.

**Conclusion:**

Enhancement patterns on a dynamic contrast CT scan of the liver are useful in the interpretation of CT images for specific diagnoses.

## Introduction

Because of the current increase in incidence of primary liver cancers, it is important to apply a systematic approach in the diagnosis and management of focal liver lesions.^1^

Key in the evaluation of focal liver lesions is a thorough clinical history and physical examination, relevant laboratory and radiological investigations, and histopathology. Histopathology is the gold standard in making the final diagnosis; however, it is not always possible, as it is invasive.^2^

Computed tomography (CT) is currently routinely used in the evaluation of the liver.^3^ Dynamic CT has been found to have high sensitivity and specificity in the diagnosis of focal liver lesions. An earlier prospective multi-institutional study performed by Vassiliades et al.^4^ compared the sensitivity of CT and high-field MRI (1.5T) in the detection of hepatic metastases. The study included 69 cancer patients. Non-contrast CT showed an overall sensitivity of 57%, dynamic CT 71% and delayed CT 72%.

Various subsequent studies showed improved performance of dynamic CT in focal liver lesions. Triphasic CT of focal liver tumours performed in Pakistan to differentiate benign from malignant lesions was found to have sensitivity and specificity of 100% and 80%, respectively. The positive predictive value (PPV) was 94.5%, negative predictive value (NPV) was 100% and diagnostic accuracy 95.5%.^5^ A systematic review by Colli et al. in patients with chronic liver disease showed the sensitivity and specificity of helical CT in the diagnosis of hepatocellular carcinoma (HCC) to be 68% and 93%, respectively.^6^ Another systematic review and meta-analysis showed 74.8% sensitivity and 95.6% specificity in the diagnosis of hepatic metastases.^7^ Sensitivity estimates for helical CT in colorectal liver metastases in another meta-analysis was 64.7%.^8^

The Liver Imaging Reporting and Data System (LI-RADS), launched in 2011, is used to standardise the reporting of dynamic imaging of the liver in patients at risk of HCC. Nodules are categorised as definitively benign (LI-RADS 1), probably benign (LI-RADS 2), indeterminate probability of being HCC (LI-RADS 3), probably HCC (LI-RADS 4) and definitively HCC (LI-RADS 5).

Liver imaging in various imaging centres in our country has no standard CT protocol. Most patients are scanned in the portovenous phase only, and this poses a challenge in the characterisation of lesions. The main reason for this is not documented; however, it is important to bring to the fore the diagnostic value of dynamic contrast CT and to avoid unnecessary extra radiation to patients who receive incomplete or inadequate studies.

Multiple studies have been conducted around the world on the role of dynamic contrast CT in characterising and distinguishing focal liver lesions. However, to the best of our knowledge, no data have been published locally, thus the purpose of this study was to compare local findings and diagnostic accuracy to studies in other parts of the world. This will help to evaluate our standard of diagnostic performance and hence initiate strategies for improvement where need be. This study aims at creating a local database on the spectrum of findings and the diagnostic performance of dynamic contrast CT imaging of the liver. The study findings will be useful to both radiologists and hepatobiliary surgeons.

## Methods

This prospective, cross-sectional study was performed at the radiology departments of the Kenyatta National Hospital and Plaza Imaging Solutions. The aims of the study were firstly to determine the liver findings on dynamic contrast CT scanning and correlate these findings with clinicopathologic findings and secondly to evaluate the diagnostic performance of dynamic contrast CT of the liver.

The sample size was determined using Fisher’s formula. Patient consent was obtained. The study included patients who were at least 18 years of age who presented to the radiology department for abdominal CT scan and who were suspected to have liver pathology (based on clinical examination and/or previous ultrasound findings) with no known primary malignancy. Patients who declined to give consent, had any contraindication to contrast media use, had a known extra-hepatic malignancy, had a known histologic diagnosis of the hepatic lesion or had inconclusive biopsy results were excluded. Sixty-one patients were recruited for the study.

A questionnaire was used to collect both qualitative and quantitative data. It recorded the patients’ demographic information, clinical history, family history and socio-economic history.

The CT scanner Somatom Definition AS 128 Slice by SIEMENS (2014) was used at Kentatta National Hospital (KNH) and the Aquilion One 640 slice by TOSHIBA (2013) was used at Plaza Imaging Solutions. Precontrast scans were done followed by contrast-enhanced triple-phase scans. Arterial phase scanning was acquired at 30–40 s following dynamic injection of the contrast agent, portovenous phase at 70 s and equilibrium/delayed phase at 180 s.

Enhancement characteristics of each lesion in each phase were evaluated by two consultant radiologists and the principal investigator. The findings were then recorded in tabulated forms.

The patients had a follow-up ultrasound-guided liver biopsy. The histological specimens were sent to Pharmcet Healthcare Laboratory and analysed by an experienced consultant pathologist. Histology results were recorded alongside the radiological findings.

Data were analysed using the SPSS program (version 20.0). The quantitative data were analysed by using descriptive statistics and presented through percentages, means, standard deviations and frequencies. The information was displayed by the use of bar charts, pie charts, tables and in prose form.

## Ethical consideration

Permission to conduct this research was obtained from Kenyatta National Hospital, University of Nairobi Ethics and Research Committee (reference number P931/12/2016).

## Results

Sixty-one patients with focal liver lesions were analysed with dynamic contrast CT scanning. Ten presented with characteristically benign lesions and were thus not biopsied. These included eight simple hepatic cysts and two haemangiomas. Fifty-one patients with a total of 62 focal liver lesions underwent biopsy. Seven patients had more than one focal lesion; however, only the most suspicious lesion was biopsied. The assumption was that all lesions were part of the same disease process.

Out of the 51 focal liver lesions, seven (13.7%) were benign and 44 (86.3%) were malignant, based on characteristic enhancement patterns. Final histopathological results showed eight (15.7%) benign and 43 (84.3%) malignant lesions. Out of the eight benign lesions, two (25%) were adenomas, two (25%) liver abscesses, one (12.5%) haemorrhagic cyst, one (12.5%) fibrosis and two (25%) focal nodular hyperplasia (FNH).

Malignant lesions were HCC (44.2%), metastatic adenocarcinoma (30.2%), cholangiocarcinoma (18.6%), metastatic squamous cell carcinoma (2.3%), gallbladder adenocarcinoma (2.3%) and non-Hodgkin’s lymphoma (2.3%). Mean lesion size was 5.8 cm with a range of 1.4 cm–17.6 cm. Benign lesions had a mean diameter of 4.7 cm while malignant lesions had a mean diameter of 6.3 cm. Metastatic lesions had a mean diameter of 4.5 cm. Different enhancement patterns were seen in the 51 biopsied lesions. The sensitivity, specificity and PPV for different enhancement patterns associated with each diagnosis are summarised in Table 1.

**TABLE 1:** Diagnoses and corresponding enhancement.

Variable	Sensitivity (%)	Specificity (%)	PPV (%)
HCC homogeneous	10.5	87.5	28.5
HCC abnormal vessels	57.9	90.6	78.6
HCC heterogeneous	31.6	78.1	46.2
FNH heterogeneous	100.0	95.9	15.4
Metastasis homogeneous	21.4	89.2	42.
Metastasis with abnormal vessels	21.4	70.3	21.4
Metastasis with incomplete ring	7.1	97.9	50
Metastasis with no enhancement	50.0	89.2	63.6
Cholangiocarcinoma heterogeneous	50.0	77.4	30.8
Cholangiocarcinoma no enhancement	25.0	77.4	18.2
Cholangiocarcinoma delayed enhancement	25.0	95.3	50.0

PPV, positive predictive value; HCC, hepatocellular carcinoma; FNH, focal nodular hyperplasia.

**FIGURE 1:**
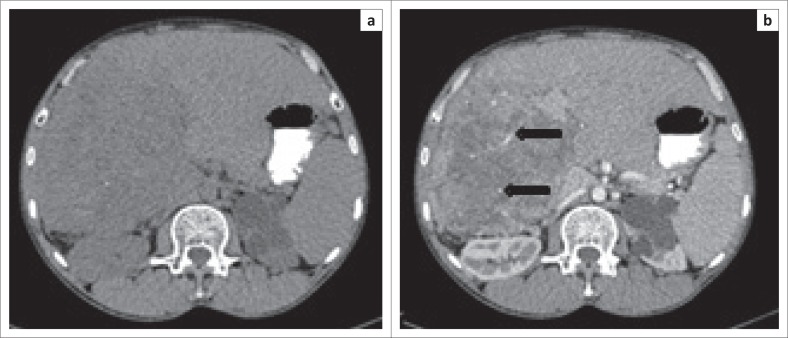
A 73-year-old man who presented with abdominal pain. Axial computed tomography scan images: (a) precontrast; (b) arterial phase shows abnormal internal vessels (black arrows).

Hepatocellular carcinoma lesions showed three patterns of enhancement: homogenous, abnormal internal vessels and heterogeneous enhancement. Abnormal internal vessel pattern (Figure 1) was most sensitive (57.9%) and specific (90.6%) with a high PPV (78.6%).

**FIGURE 2:**
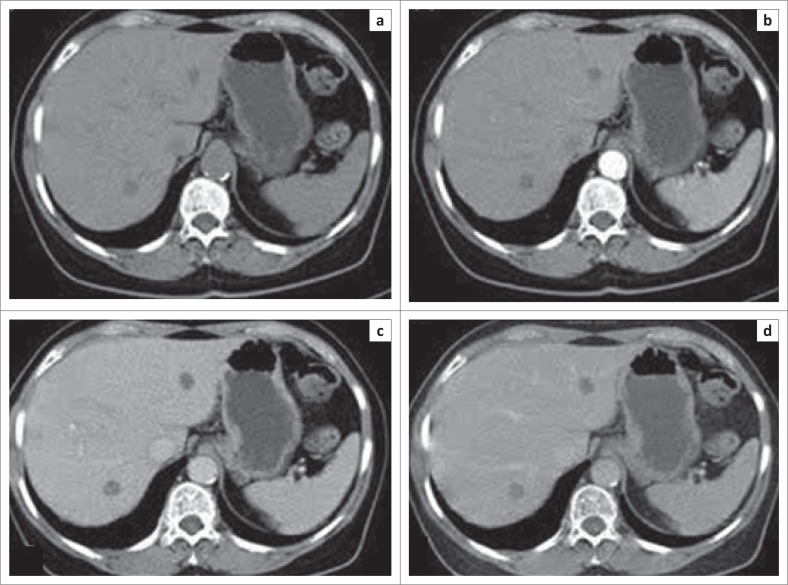
A 70-year-old man who presented with abdominal pain with no known primary malignancy. Axial computed tomography scan images of biopsy proven metastatic adenocarcinoma showing: (a) hypo-attenuation in the precontrast phase and no enhancement in (b) arterial, (c) portovenous and (d) delayed phase.

**FIGURE 3:**
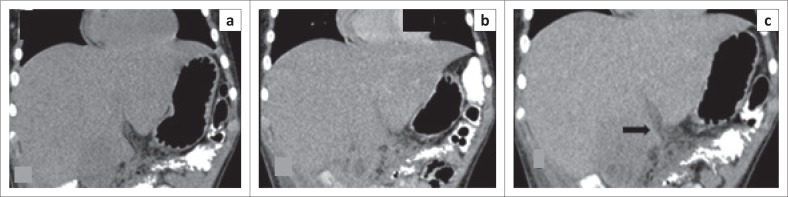
A 36-year-old female who presented with a long history of intermittent fever. Coronal computed tomography scan images of an abscess in segment V of the liver (a) precontrast, (b) arterial phase and (c) portovenous phase showing incomplete ring enhancement (arrow).

Heterogeneous enhancement of FNH had 100% sensitivity and 95.9% specificity but had a low PPV (15.4%). The non-enhancing pattern of metastasis (Figure 2) exhibited sensitivity of 50% and specificity of 89.2% with a PPV of 63.6%. An abscess showed incomplete ring enhancement as seen in Figure 3.

**FIGURE 4:**
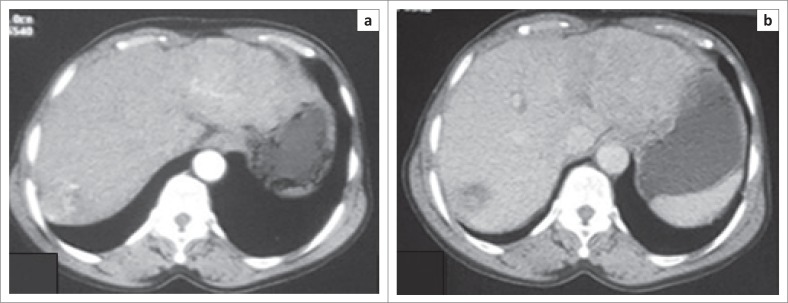
A 54-year-old male with hepatocellular carcinoma in segment VII of the liver who presented with right upper-quadrant pain. Axial computed tomography scans showing: (a) arterial phase enhancement and (b) rapid washout in the portovenous phase.

Delayed enhancement of cholangiocarcinoma (Figure 4) showed 25% sensitivity and 95.3% specificity with a PPV of 50%. Thirteen of the HCC lesions demonstrated rapid arterial washout with a sensitivity of 68.4%, specificity of 87.5% and a PPV of 72.2%.

Of the histologically proven lesions, 35 (57.4%) were in males and 26 (42.6%) in females. Malignancies were seen in 65% of males and benign lesions in 75% of females. Chronic liver disease resulting from hepatitis B virus (HBV) was seen in one (5%) of the HCC patients (*p* = 0.373). Chronic alcohol use was noted in 65% of patients with HCC (*p* = 0.046). No patient with HCC confirmed HIV infection. Seven (36.8%) of the 19 patients with HCC reported a history of smoking (*p* = 0.247). Further, 75% of patients with cholangiocarcinoma had a history of chronic alcohol use (*p* = 0.119) and 50% history of smoking (*p* = 0.192). Both patients with adenoma had a history of oral contraceptive use (7 and 10 years’ duration).

Dynamic CT diagnosed 44 (86.3%) malignant and seven (13.7%) benign lesions based on characteristic enhancement patterns. On later histological evaluation, 43 (84.3%) were malignant and eight (15.7%) were benign. Thus, there were 40 true positive, four false positive, four true negative and three false negative results reported on CT-based assessment of liver lesions. Dynamic CT was found to have a sensitivity of 93%, specificity of 50%, PPV of 91%, NPV of 57% and diagnostic accuracy of 95.5% in differentiating benign from malignant liver lesions.

The LI-RADS categories of the lesions are summarised in Table 2.

**TABLE 2:** Liver Imaging Reporting and Data System categories of the 61 liver lesions.

LI-RADS category	Number of lesions	Final histological diagnosis					
		HCC		Other malignancy		Benign lesions	
		*N*	%	*N*	%	*N*	%
1	10	0	-	0	-	10	-
2	4	0	-	1	25	3	75
3	4	0	-	3	75	1	25
4	6	0	-	4	75	2	25
5	24	18	75	6	25	0	-
OM	13	1	7.7	10	76.9	2	15.4

LI-RADS, Liver Imaging Reporting and Data System; HCC, hepatocellular carcinoma; OM, other malignancy.

Lesions classified as LI-RADS 1 had characteristic benign findings, as either simple cysts or haemangiomas and were thus not biopsied. One (25%) of the LI-RADS 2 lesions was a metastatic lesion, while the remaining lesions (75%) were two adenomas and a haemorrhagic cyst. Three (75%) of the LI-RADS 3 lesions were metastases, and one (25%) was an abscess. Four (75%) of the lesions classified as Category 4 were malignant (three cholangiocarcinoma and one non-Hodgkin’s lymphoma). Two (25%) of the lesions classified as Category 4 were FNH. Eighteen (75%) of the LI-RADS 5 lesions were HCC and six (25%) were other malignancies (one cholangiocarcinoma and five metastases). One (7.7%) of the lesions classified as category other malignancy (OM) was HCC, 10 (76.9%) were other malignancies (four cholangiocarcinoma, five metastases, one gallbladder adenocarcinoma) and two (15.4%) were benign (tuberculous abscess, fibrosis). The diagnostic accuracies for HCC diagnosis of the LI-RADS criteria are summarised in Table 3.

**TABLE 3:** Diagnostic performance of hepatocellular carcinoma diagnosis using Liver Imaging Reporting and Data System 4 and 5 categories.

Diagnostic criteria	Number of nodules				Diagnostic performance (%)			
	TP	FN	FP	TN	Sensitivity	Specificity	PPV	NPV
LI-RADS Category 5	18	1	6	26	94.7	81.3	75	96.3
LI-RADS Categories 4 and 5	18	1	12	20	94.7	62.5	60	95.2

LI-RADS, Liver Imaging Reporting and Data System; TP, true positive; TN, true negative; FP, false positive; FN, false negative; PPV, positive predictive value; NPV, negative predictive value.

## Discussion

Our study documented 84.3% malignant lesions, which is comparable to a retrospective study by Matilde et al.^9^ that excluded simple cysts and showed 87% of the lesions to be malignant. Metastases were the most common malignant finding at 53%, followed by HCC at 31%. This was unlike our study, which showed HCC to be the most common malignant lesion at 44.2%.

The mean lesion size of 5.8 cm was higher than in the study by Matilde et al.,^9^ which found 4.9 cm, and a similar study in Pakistan by Hafeez et al.,^5^ which found 3.4 cm. Metastatic lesions in this study were larger in size (mean 4.5 cm) compared to those in a study by Soyer et al., which had a mean lesion size of 2.2 cm.^10^ These larger sizes could be explained by the health-seeking behaviour in our population, where patients present at a late stage of disease. No differences were noted between the patients at Kenyatta National Hospital and those at Plaza Imaging Solutions.

Hepatocellular carcinoma showed three patterns of enhancement: homogenous, abnormal internal vessels and heterogeneous enhancement. The abnormal internal vessel pattern was most specific (90.6%) and showed a high PPV (78.6%). Heterogeneous enhancement of FNH had 95.9% specificity but had a low PPV (15.4). Matilde et al. showed the abnormal vessels of HCC to have a specificity of 98% and a PPV of 90%.^9^ Rapid washout showed a specificity of 87.5% and PPV of 72.2% in the diagnosis of HCC. This study showed 68.4% of HCC had rapid washout. Lee et al. found arterial hyperenhancement in 86% and 78% of HCC lesions by the two observers, respectively, and 76% showed rapid washout.^11^

Studies by Ferdey et al.^12^ and Lancomis et al.^13^ showed 75% and 74%, respectively, of hyperattenuation of cholangiocarcinoma on delayed images. The hyperattenuating areas in cholangiocarcinoma on delayed phase images seem to be related to the large amount of interstitial space in the fibrous stroma of the tumour.^14^ Delayed enhancement of cholangiocarcinoma showed a specificity of 95.3% and PPV of 50% in our study.

Various studies have shown most metastatic liver lesions to be hypovascular. The non-enhancing pattern of metastasis exhibited a specificity of 89.2% and PPV of 63.6% in this study. The non-enhancing pattern of metastasis demonstrated a specificity of 96% and a PPV of 75% in the study by Matilde et al.^9^ In a retrospective study by Leslie and colleagues, 90% of metastases showed hypodense enhancement on arterial phase.^15^ Precontrast imaging allowed depiction of all hepatic metastases (sensitivity, 100%), compared to 66% in the study by Soyer et al.^10^

Malignant lesions were more frequent in men (65%), while benign lesions were mostly in women (75%). Kerlin et al. also demonstrated malignant lesions to be more common in males.^16^ Hepatic adenomas and FNH were exclusively found in females. Baum et al. were the first to demonstrate the relationship of hepatic adenomas and oral contraceptive use.^17^ In a study by Kerlin et al. it was observed that patients with hepatic adenoma were young and 91% were female. They observed that 89% of them had used oral contraceptives.^16^ In this study both of the patients with adenoma reported oral contraceptive use. Hepatocellular carcinoma and cholangiocarcinoma occurred predominantly in males (M:F 16:3 and 3:1, respectively).

There was a significant correlation between the history of alcohol intake and HCC in this study (*p* = 0.046). Heavy alcohol consumption has been shown to increase the risk of HCC.^18^ Globally, 78% of HCC is attributable to HBV (53%) or hepatitis C virus (HCV) (25%), according to a study by Perz et al.^19^

Sampling errors resulting from liver biopsy technique were beyond the scope of this study. Only patients with conclusive histology findings were included. Although high diagnostic accuracy (86%) was noted in our results, we had four false positive results. Three of these lesions were labelled as malignant because of hypervascularity and proved to be FNH. One lesion was hypovascular and proved to be a tuberculous abscess. Higher diagnostic accuracy of 95% was recorded in a similar study by Hafeez et al.^5^ This study demonstrated dynamic CT scan to have a sensitivity of 93%, specificity of 50% and PPV of 91% in differentiation of benign and malignant liver lesions. Possible causes of false negative cases could be inaccurate timing of the different phases of enhancement.

Considering only LI-RADS 5 as conclusive for HCC diagnosis, our study missed one out of the 19 HCC lesions (specificity = 81.3%). However, if both LI-RADS 4 and 5 are combined as definitely indicating the diagnosis of HCC, the specificity decreased to 62.5% but the sensitivity remained the same at 94.5%. A study by Trillaud et al.^20^ on characterisation of liver nodules using MRI showed improved sensitivity and reduced specificity when combining LI-RADS 4 and 5. We confirmed that lesions classified as LI-RAD 5 that show arterial phase hyperenhancement and washout at CT should be considered as HCC, as documented in the latest LI-RADS version.^21^ There is benefit in differentiating LI-RAD Category 4 and 5 lesions to improve diagnostic accuracy.

## Conclusion

Dynamic contrast CT scan has high diagnostic accuracy in focal liver lesions. Various lesions show characteristic enhancement patterns that are useful in the interpretation of CT images for specific diagnoses, emphasising the need for national standardisation of protocols for liver evaluation.

### Limitations of the study

Only one lesion per patient was biopsied, with the assumption that all lesions were the same disease process. A larger sample size and additional local studies are necessary to strengthen our findings. Some of the patients who were candidates for this study were lost to follow-up, either because of death or because of financial constraints. Sampling errors resulting from liver biopsy technique were beyond the scope of this study.
